# A Flexible NO_2_ Gas Sensor Based on Single-Wall Carbon Nanotube Films Doped with a High Level of Nitrogen

**DOI:** 10.3390/molecules27196523

**Published:** 2022-10-02

**Authors:** Xiao-Han Tian, Tian-Ya Zhou, Yu Meng, Yi-Ming Zhao, Chao Shi, Peng-Xiang Hou, Li-Li Zhang, Chang Liu, Hui-Ming Cheng

**Affiliations:** 1 Shenyang National Laboratory for Materials Science, Institute of Metal Research, Chinese Academy of Sciences, Shenyang 110016, China; 2 School of Materials Science and Engineering, University of Science and Technology of China, Hefei 230026, China; 3 Faculty of Materials Science and Engineering/Institute of Technology for Carbon Neutrality, Shenzhen Institute of Advanced Technology, Chinese Academy of Sciences, Shenzhen 518055, China

**Keywords:** single-wall carbon nanotube, nitrogen doping, flexible sensor

## Abstract

Carbon nanotubes (CNTs) are considered a promising candidate for the detection of toxic gases because of their high specific surface area and excellent electrical and mechanical properties. However, the detecting performance of CNT-based detectors needs to be improved because covalently bonded CNTs are usually chemically inert. We prepared a nitrogen-doped single-wall CNT (SWCNT) film by means of gas-phase fluorination followed by thermal annealing in NH_3_. The doped nitrogen content could be changed in the range of 2.9–9.9 at%. The N-doped SWCNT films were directly used to construct flexible and transparent gas sensors, which can work at a low voltage of 0.01 V. It was found that their NO_2_ detection performance was closely related to their nitrogen content. With an optimum nitrogen content of 9.8 at%, a flexible sensor had a detection limit of 500 ppb at room temperature with good cycling ability and stability during bending.

## 1. Introduction

NO_2_ is one of the major atmospheric pollutants as a byproduct of coal combustion and petroleum refining. It causes acid rain, photochemical smog, and irritation in the human respiratory system. Therefore, the development of flexible sensors capable of sensitively detecting NO_2_ is highly desired in the fields of wearable electronics [1,2,3], healthcare [4,5] and military detection [6]. A high-performance sensing material should be robust enough to sustain stable electrical performance over medium to long periods of time, yet sensitive enough to detect small changes in the surrounding environment. Carbon nanotubes (CNTs) are considered a promising candidate because of their large surface area and unique electrical and mechanical properties [7]. In particular, single-wall CNTs (SWCNTs) can be semiconducting or metallic depending on their chirality and have a large specific surface area that provides numerous adsorption sites. The adsorption of NO_2_ gas onto pure SWCNTs without any chemical functionalization has been shown to produce a sensing response [8,9,10,11]. When target gases are adsorbed on the surface of a SWCNT with an applied voltage, the current increases (or decreases) because of the change in the concentration of hole carriers [12]. Because of this, gas sensors based on semiconducting SWCNTs have a higher response [13,14,15]. However, the difficulty in obtaining high-purity semiconducting SWCNTs has limited their commercial applications.

High-quality SWCNT networks show potential for the fabrication of flexible, sensitive, low-power gas sensors as wearable electronics [12,16,17,18]. However, due to the inertness of the sp^2^ hybridized SWCNTs, doping or functionalization is generally required to introduce active sites in CNTs to improve their sensing performance. There have been several approaches to increase the sensitivity of SWCNT films to gases, and these can be classified into introducing nanoparticles [19,20,21], noncovalent functionalization [22,23,24] and covalent functionalization [25,26]. Nitrogen doping, a covalent functionalization method, is considered an efficient way to controllably change the structure and properties of CNTs. It can be realized in two different ways, i.e., doping during synthesis [27,28,29] and a doping post-treatment [30,31]. Compared with the former, the latter can achieve a higher doping level, and the range of doping sources is broader. To obtain a high doping level, vacancies are usually created in the graphitic lattice of CNTs, resulting in the destruction of their original structure. Because of this, a major challenge is to develop an efficient doping method that produces a high level of nitrogen, while simultaneously retaining the flexibility and structural integrity of the CNT network.

With the coming of the Internet of Things (IoT) era, the development of high-performance portable and wearable gas sensors able to work at room temperature has attracted great research interest. As an important part of IoT devices, a new generation of gas sensors requires low power consumption ensuring potential use in smart phones and wireless sensor platforms [32]. As far as we know, this issue has rarely been addressed.

We prepared a nitrogen-doped single-wall CNT (SWCNT) film by gas-phase fluorination followed by NH_3_ thermal annealing. A high nitrogen content of up to 9.9 at% was achieved, and most of the doped nitrogen was in the form of pyridine N, which is highly active for NO_2_ sensing [10,27]. A gas sensor consisting of a nitrogen-doped SWCNT film on a PET substrate showed excellent flexibility and a high light transmittance of 86%, which has promise for use in portable or wearable detection devices for low concentrations of NO_2_.

## 2. Experimental Section

### 2.1. Preparation of Flexible and Transparent SWCNT Films

Flexible and transparent SWCNT films were prepared by a floating catalyst chemical vapor deposition (FCCVD) method [33]. The growth temperature was 1100 °C, and hydrogen was used as a carrier gas. A solution of toluene (10 g), ferrocene (0.3 g), and thiophene (0.045 g) acting as carbon source, catalyst precursor, and growth promoter, respectively, was injected into the reactor by a syringe pump at a rate of 0.24 mL/h, while 11 sccm of C_2_H_4_ gas as a carbon source was also introduced. SWCNT films were collected on a porous cellulose filter membrane (0.45 μm diameter pores; collection area, 100 mm × 100 mm) placed at the outlet of the reactor. As shown in Figure S1, the as-collected SWCNT film shows a good uniformity.

### 2.2. Preparation of N-Doped SWCNT Films

#### 2.2.1. Fluorination of SWCNT Films

The prepared flexible and transparent SWCNT films were transferred onto a Teflon frame of size 1.2 cm × 1.6 cm, and this together with a certain amount of XeF_2_ were placed in a 300 mL Teflon container. The container was placed in an oven and heated to 100 °C where it was kept for 1, 2, 4, or 8 h to obtain fluorinated SWCNT (F-SWCNT) films. The detailed fluorination parameters of 6 samples are summarized in Table S1. We used sample #4 to elucidate the structural characteristics in the following work unless otherwise mentioned.

#### 2.2.2. Synthesis of N-Doped SWCNT Films by Ammoniation

The prepared F-SWCNT film was placed in a quartz boat and then put into a tubular furnace and heated to 500 °C, and then kept in an 80 sccm ammonia gas flow for 1 h. Finally, the furnace was naturally cooled to room temperature under the protection of an argon flow. The sample obtained is denoted N-SWCNT.

### 2.3. Fabrication of Flexible N-SWCNT-Based Sensors

We fabricated a two-electrode flexible sensor using the prepared N-SWCNT film. Briefly, gold stripes with a separation of 0.8 cm were deposited on a PET substrate by magnetron sputtering for use as the electrodes. The N-SWCNT film was then transferred onto the PET substrate covered by the Au electrodes. Ethanol was then dripped on and spread over the film to cause the N-SWCNT film to make tight contact with the gold electrodes. Figure S2 shows typical optical images of the constructed N-SWCNT sensor, from which we can see that the device is highly flexible and transparent.

## 3. Results and Discussion

### 3.1. Characterization of the N-SWCNT Films

**Figure 1:**
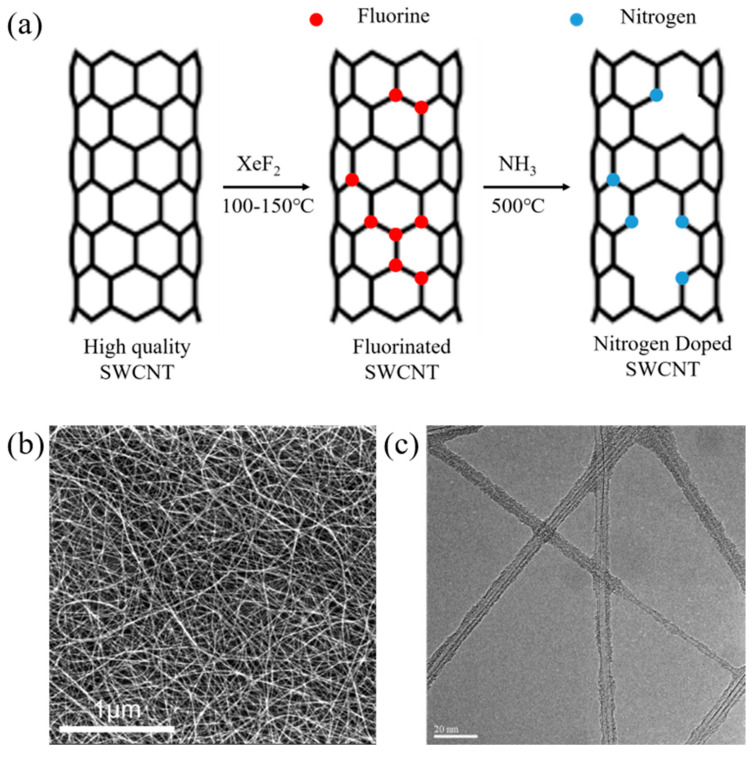
(**a**) Schematic showing the N–doping process of SWCNTs. (**b**) Typical SEM image of the as-prepared N–SWCNT film. (**c**) Typical TEM image of the N–SWCNTs.

The nitrogen-doping process of the SWCNT films is schematically shown in Figure 1a. When the SWCNTs are exposed to F_2_ produced by the decomposition of XeF_2_, some C-F bonds are formed in the SWCNT lattice, and an F-SWCNT film was obtained (Figure 1a). When this film was heated at 500 °C in an ammonia atmosphere, defluorination occurred due to the instability of the C-F bonds, and vacancies were formed in the lattice structure of SWCNTs [30,31]. In the ammonia atmosphere, nitrogen atoms occupied the vacant lattice sites in the SWCNTs, yielding a N-SWCNT film.

**Figure 2:**
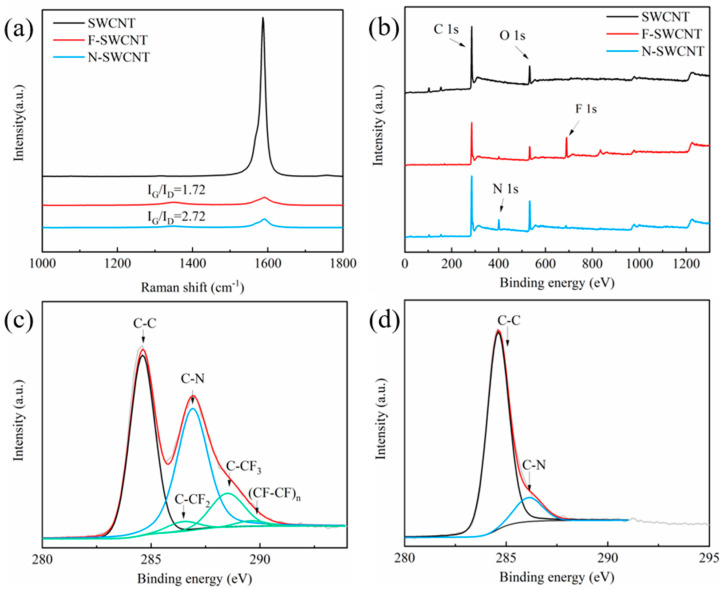
(**a**) Raman and (**b**) XPS spectra of SWCNT, F–SWCNT and N–SWCNT films. (**c**) C 1 s curves of the F–SWCNT film. (**d**) C 1 s curves of the N–SWCNT film.

Figure 1b shows a typical scanning electron microscope (SEM) image of the N-SWCNT film. Numerous randomly entangled filaments (SWCNT bundles) are observed, which provide well-connected electron transport paths in the film. Compared to the original SWCNT and the F-SWCNT films (Figure S3a,b), no obvious morphology change was detected (Figure S3b). Figure 1c is a typical TEM image of the N-SWCNTs, showing small bundles with a mean diameter of ~10 nm, confirming that the fluorination and ammonization processes do not change the bundle structure and exposed surface area, compared to the original SWCNT samples (Figure S4a,b). During annealing, N atoms occupy the vacancies left by defluorination to form a high density of pyridinic active sites that stimulate the recovery of the graphitic lattice structure. The structural recovery of the N-SWCNT is confirmed by the Raman spectra shown in Figure 2a. It can be seen that the I_G_/I_D_ value, which is a benchmark for evaluating the crystallinity of SWCNTs, increased from 1.72 to 2.72 after the heat treatment. A good crystallinity of the N-SWCNT enables fast electron transport, which improves the gas sensing performance.

**Figure 3:**
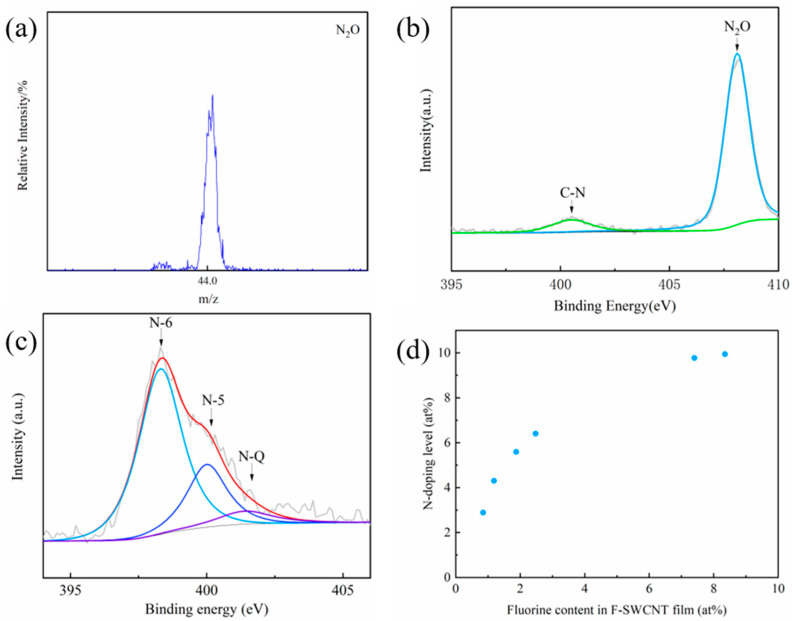
(**a**) SIMS spectrum of a F–SWCNT film showing the existence of N_2_O molecules. (**b**) N 1 s XPS spectra of a F–SWCNT film. (**c**) N 1 s XPS spectra of a N–SWCNT film. (**d**) Dependence of the N–doping content on the degree of fluorination of the SWCNTs.

The surface elemental composition and bonding configuration of the SWCNTs were investigated using X-ray photoelectron spectroscopy (XPS, Figure 2b). A strong F 1 s peak was detected in the F-SWCNT sample, and the F content reached 8.35 at% (Table S1). Compared to the original SWCNT film, the high-resolution C 1 s spectra of the F-SWCNT film show obvious additional peaks at 286.4 eV, 288.4 eV, and 289.6 eV, which are assigned to C-F bonding (Figure 2c). After defluorination, the C-F peak disappeared, as shown in Figure 2b. Unexpectedly, a nitrogen signal was also detected in the F-SWCNT film (Figure 2b). The adsorbed N_2_O can be desorbed with ~200 °C heat treatment, as shown in Figure 2d. Secondary ion mass spectroscopy (SIMS) (Figure 3a) showed that the nitrogen in the F-SWCNT films was mainly N_2_O absorbed on the F-SWCNT bundles, which is consistent with the XPS measurements (Figure 3b).

After defluorination at 500 °C, the F 1 s peak disappeared while an obvious N 1 s peak was detected. The N 1 s spectrum of the N-SWCNT film shown in Figure 3c was deconvoluted into three peaks of pyridinic N (398.6 eV, N-6), pyrrolic N (400.3 eV, N-5), and graphitic N (401.1 eV, N-Q). Furthermore, auxiliary energy dispersive X-ray spectroscopy (EDS) elemental analysis (Figure S5) shows that nitrogen is homogeneously distributed in the SWCNT bundle. The content of pyridinic N in the N-SWCNT film (Figure 3c) was then calculated to be as high as 68.8% (Table S2). Furthermore, the N 1 s peak intensity of the N-SWCNT films increased with an increased degree of fluorination (Figure S6). The dependence of the N-doping level on the fluorine content of the SWCNT films is shown in Figure 3d. It can be seen that the content of doped nitrogen can be controlled over a wide range of 2.9~9.9 at% by changing the degree of fluorination.

### 3.2. N-SWCNT Film Based NO_2_ Sensor

**Figure 4:**
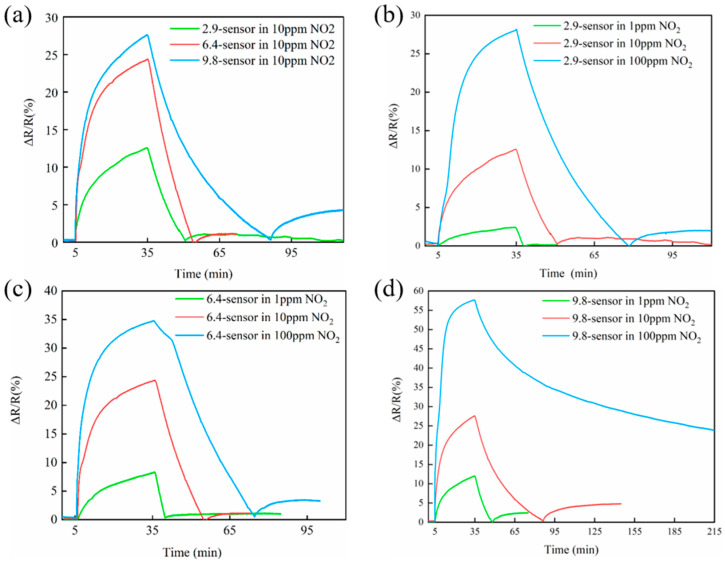
(**a**) Responsivity of the sensors exposed to 10 ppm NO_2_. (**b**–**d**) Sensing performance of the sensors exposed to different concentrations of NO_2_.

We constructed gas sensors using the N-SWCNT films with N contents of 2.9 at%, 6.4 at%, and 9.8 at%. For simplicity, the resulting sensors are, respectively named 2.9-sensor, 6.4-sensor, and 9.8-sensor. The sensing measurements were conducted using a DGL-Ⅲ gas distribution system, consisting of a chamber with a separate gas inlet and outlet. Mass flow controllers were used to control the flow rates, and argon was used as the carrier gas [15] The relative change in the resistance of the sensors and NO_2_ concentration was monitored by a CGS-MT mini-multi-functional probe station. The responsivity of the sensors is defined as the relative change in resistance. We first investigated the responsivities of N-SWCNT-based sensors with different N contents after exposure to 10 ppm NO_2_ for 30 min at 90 °C, followed by desorption of the N using UV illumination in an argon atmosphere. The sensors were tested at constant voltages of 0.2 V or 0.01 V. As shown in Figure S7 and Figure 4a, the responsivity of the sensors constructed with N-doped SWCNTs is much higher than that of pure SWCNTs, and it increases with the increase in N-doping content. The improved sensing performance can be attributed to the enhanced charge transfer induced by NO_2_ interactions, which is closely related to the density of N active sites [34,35].

The 9.8-sensor had a high responsivity of 27.7% upon exposure to 10 ppm NO_2_, which is among the best of previously reported values [12,16]. As shown in Figure 4a the recovery time of the sensors increased with the N content of the SWCNT film. It also increased 1.2 times when the N-doped content increased from 2.9 at% to 6.4 at%. It took 53 min for the 9.8-sensor to recover. These results indicate that the NO_2_ molecules have strong chemical bonding with the pyridinic/pyrrolic active sites of the N-doped SWCNT films [10,27]. The influence of NO_2_ concentration on the reversibility of the sensors was also tested. As shown in Figure 4b–d, the relative changes in the resistance of the three sensors increased with increasing NO_2_ concentration. Furthermore, a similar recovery behavior (Figure 4b–d) was observed except for the 9.8-sensor (Figure 4d) exposed to 100 ppm NO_2_, where desorption was not complete after 3 hours, even with the aid of UV light irradiation.

**Figure 5:**
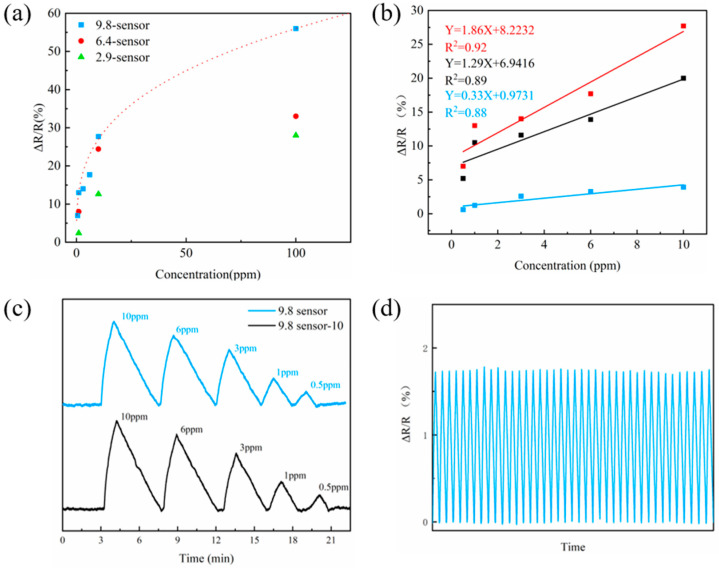
(**a**) Responsivity vs. concentration plot of the three sensors exposed to different concentrations of NO_2_. (**b**) Sensitivity of the 9.8-sensor (red line), 9.8-sensor after 30 bending (black line), and 9.8-sensor-upon-quick-cycling (blue line) exposed to 0.5–10 ppm NO_2_. (**c**) Sensing performance of 9.8-sensor and 9.8-sensor-10 exposed to different concentrations of NO_2_. (**d**) Cycling performance of 9.8-sensor-10 in 1 ppm NO_2_.

Sensitivity is also a very important parameter for real applications. We show the responses of the three sensors upon exposure to NO_2_ with different concentrations in Figure 5. As shown in Figure 5a, the sensitivity increased with the N-doping level. The 9.8-sensor showed the highest responsivity and sensitivity, with a detection limit of 500 ppb (Figure S8). We also tested the bending stability to prove that the gas senor can be used as a flexible device. The 9.8-sensor was bent into a roll with a radius of curvature of 2 mm for 30 times (Figure S9a). As shown in Figure S9b,c, the total relative change in the resistance was only 0.067 % when the sensor was bent into a curve with a radius of curvature of 4.5 mm. As shown in Figure 5b, the sensor had similar responses before and after bending. The responsivity and sensitivity only decreased slightly, which shows the excellent stability of our gas sensor during bending (Figure S9). Furthermore, the 9.8-sensor after bending had a high sensitivity of 1.29 over 0.5 ppm to 10 ppm NO_2_ exposure, which is 2.6 times higher than that reported in the literature [16].

The rapid cycling performance of the 9.8-sensor with the highest responsivity and sensitivity was measured by exposure to various concentrations of NO_2_ (from 10 ppm to 500 ppb) at room temperature. We performed quick-cycling experiments by exposing 9.8-sensor to NO_2_ gas for 1 min, which was then completely desorbed with the aid of UV irradiation. As shown in Figure 5c, the sensor showed a gradually decreased response with the decrease in NO_2_ concentration. Furthermore, this characteristic is well maintained even when the 9.8-sensor was placed in air for 10 months (9.8-sensor-10), verifying a good stability. We further measured the quick-cycling ability of 9.8-sensor-10 in 1 ppm NO_2_ for 40 cycles. As shown in Figure 5d, the 9.8-sensor-10 shows a stable performance over 40 cycles without observable loss of responsivity.

Our sensor also showed a good light transmittance of 86% under a 550 nm laser and no significant baseline drift or performance decay after continuous measurement, which could be very important in the design of wearable chemical sensors for practical applications.

## 4. Conclusions

We have prepared N-SWCNT films with nitrogen doping up to 9.9 at% by gas-phase fluorination followed by thermal annealing in NH_3_. Flexible and transparent NO_2_ gas sensors were constructed using the N-SWCNT films. The detectors had the ability to detect extremely low NO_2_ concentrations of ppb level. We attribute this low detection limit to the high content of pyridinic- and pyrrolic-N active sites introduced in the N-SWCNT film by nitrogen doping. With a combination of low power consumption (operated at 0.01 V), high transparency and flexibility, our SWCNT film-based sensors have great potential for use in various portable sensing devices.

## Data Availability

Not applicable.

## Supplementary Materials

The following supporting information can be downloaded at: https://www.mdpi.com/article/10.3390/molecules27196523/s1, Figure S1: Typical optical image of a flexible and transparent SWCNT film loaded on a filter; Figure S2: Optical images of the fabricated N-SWCNT film-based flexible sensors; Figure S3: SEM images of (a) pure SWCNT and (b) F-SWCNT films; Figure S4: TEM images of (a) pure SWCNT and (b) F-SWCNT films; Figure S5: EDS elemental (C, N, and O) maps of a SWCNT bundle; Figure S6: XPS spectra of N-SWCNT films with different doped N contents; Figure S7: Sensing performance of pure SWCNTs upon exposure to NO_2_ with different concentrations; Figure S8: Responsivity of the 9.8-sensor exposed to 0.5 ppm NO_2_; Figure S9: (a) The sensor was bent into a roll with a radius of curvature of 2 mm for 30 times. (b,c) Performance measurement of the sensor after 30 cycles of bending; Table S1: Parameters of the fluorination process and the F-doping contents; Table S2: The contents of pyridinic N (N-6), pyrrolic N (N-5) and graphitic N (N-Q) in N-SWCNT samples calculated from their deconvoluted peak areas.
