# Supplementary material for: A Flexible NO2 Gas Sensor Based on Single-Wall Carbon Nanotube Films Doped with a High Level of Nitrogen
Source: Molecules. 2022 Oct 2;27(19):6523. doi: 10.3390/molecules27196523 (PMC9573668; doi:10.3390/molecules27196523)
Supplement: Supplementary file 1 [file molecules-27-06523-s001.zip › molecules-1896830-supplementary.pdf]

Supporting information

A flexible NO_2 gas sensor based on single-wall carbon nanotube films doped with a high-level of nitrogen.

Xiao-Han Tian[1,2], Tian-Ya Zhou[1,2], Yu Meng[1,2], Yi-Ming Zhao[1,2], Chao Shi[1], Peng-Xiang Hou[1,2], Li-Li Zhang[1,2], Chang Liu[1,2], Hui-Ming Cheng[1,3]

1 Shenyang National Laboratory for Materials Science, Institute of Metal Research, Chinese Academy of Sciences, Shenyang 110016, China

2 School of Materials Science and Engineering, University of Science and Technology of China, Hefei 230026, China

3 School of Materials Science and Engineering, Institute of Technology for Carbon Neutrality, Shenzhen Institute of Advanced Technology, Chinese Academy of Sciences, Shenzhen, 518055, P.R. China.

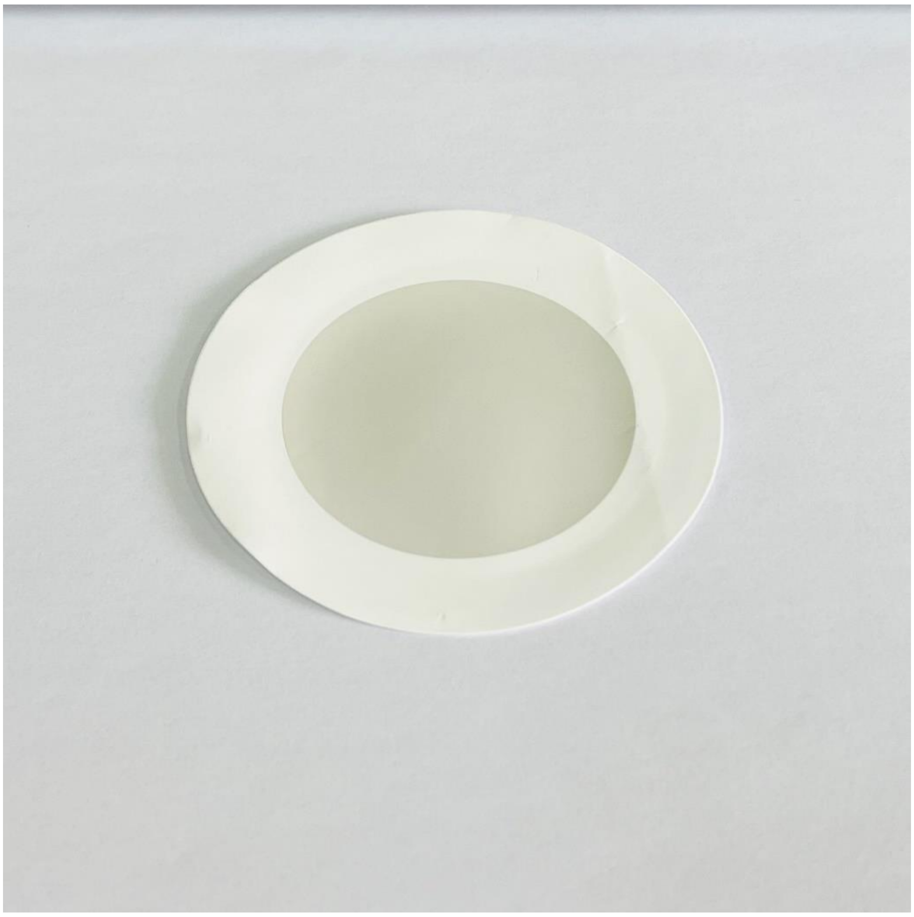

Figure S1. Typical optical image of a flexible and transparent SWCNT film loaded on a filter.

(a) (b)

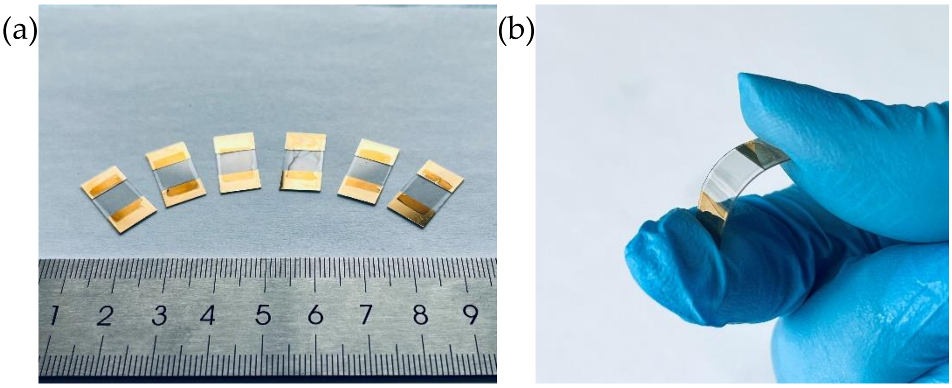

Figure S2. Optical images of the fabricated N-SWCNT film-based flexible sensors.

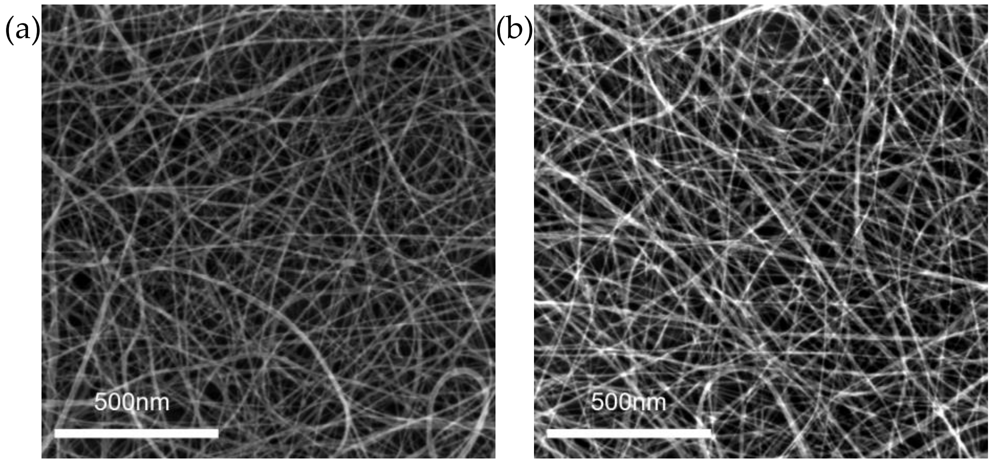

Figure S3. SEM images of (a) pure SWCNT and (b) F-SWCNT films.

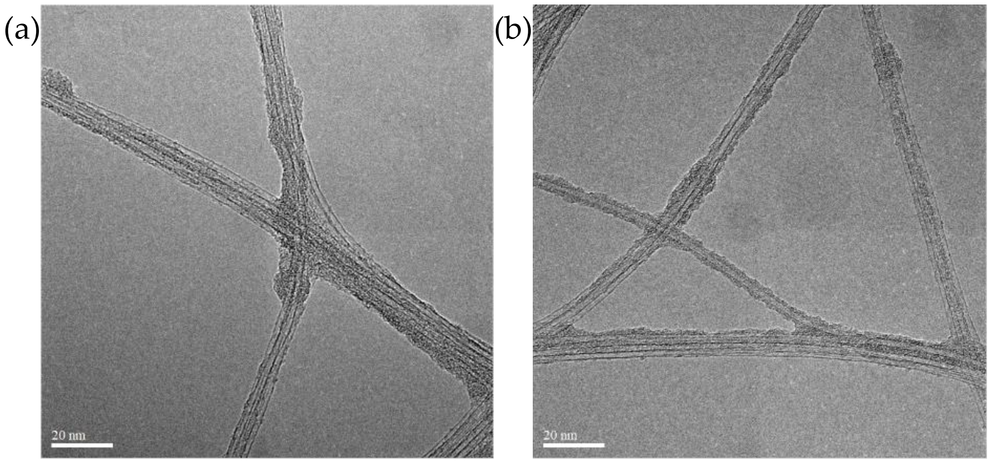

Figure S4. TEM images of (a) pure SWCNT and (b) F-SWCNT films.

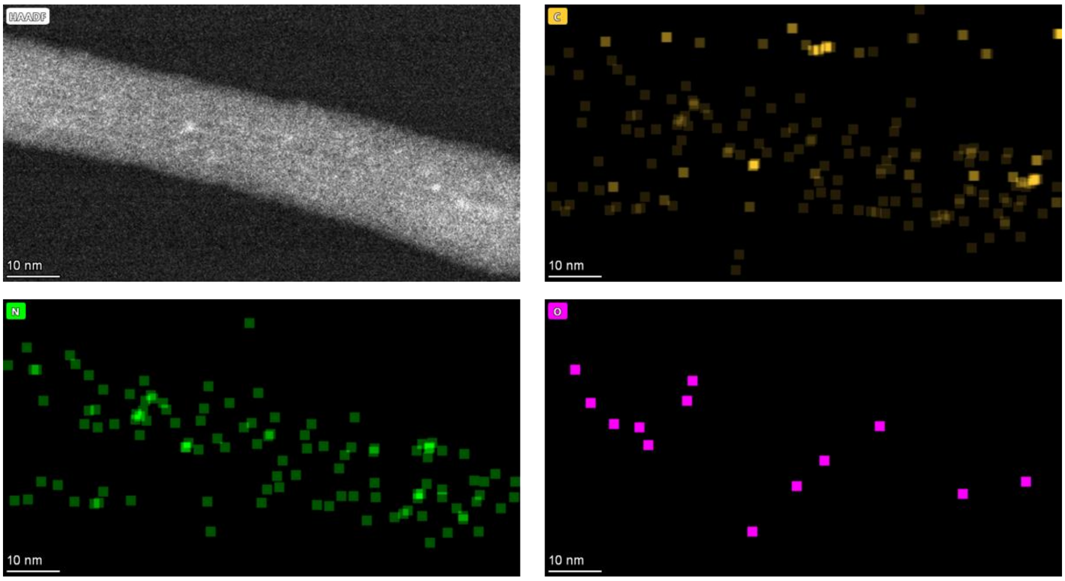

Figure S5. EDS elemental (C, N, and O) maps of a SWCNT bundle.

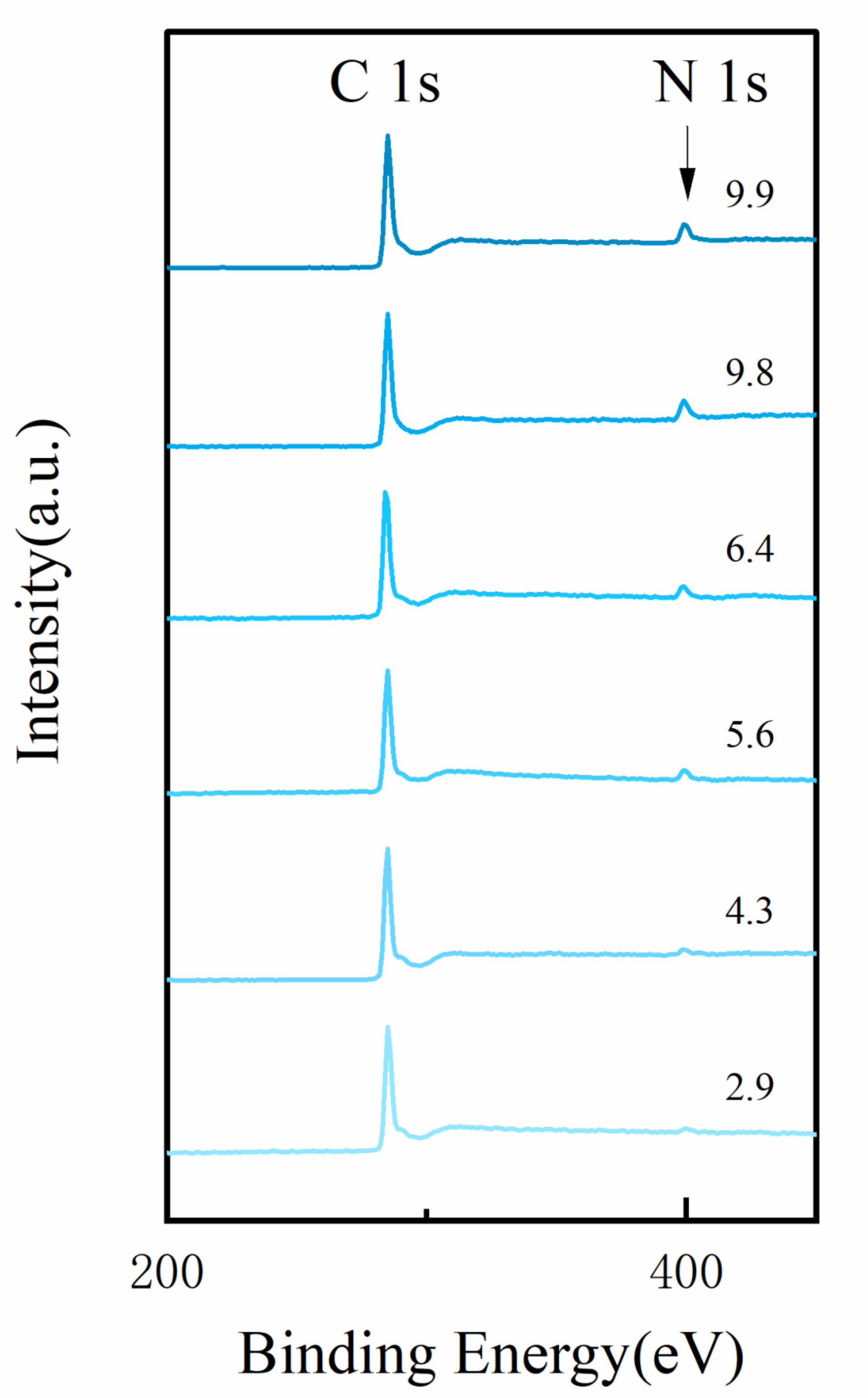

Figure S6. XPS spectra of N-SWCNT films with different doped N contents.

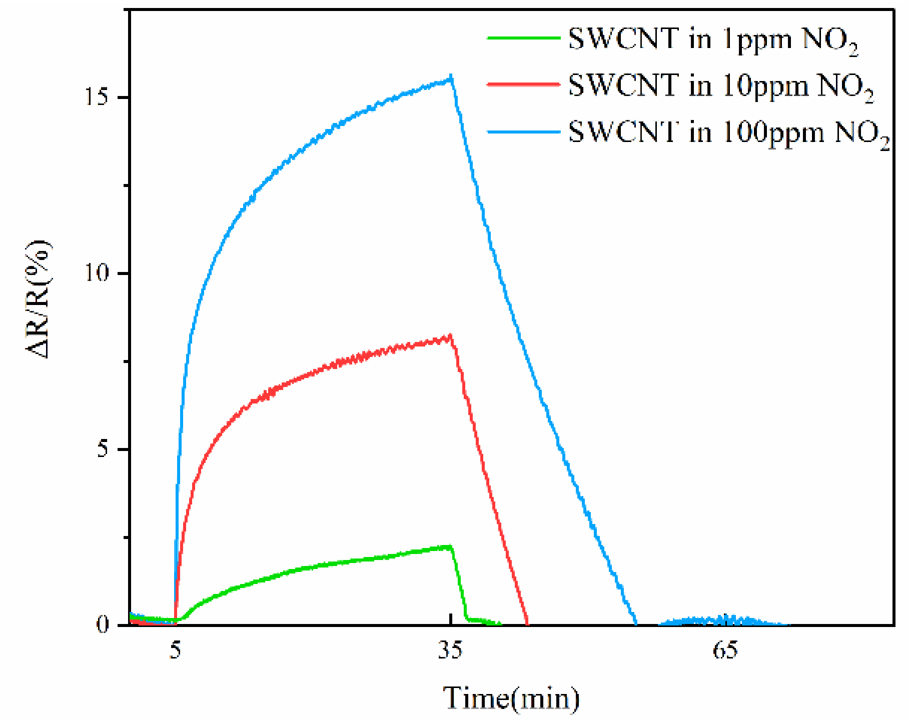

Figure S7. Sensing performance of pure SWCNTs upon exposure to NO2 with different concentrations.

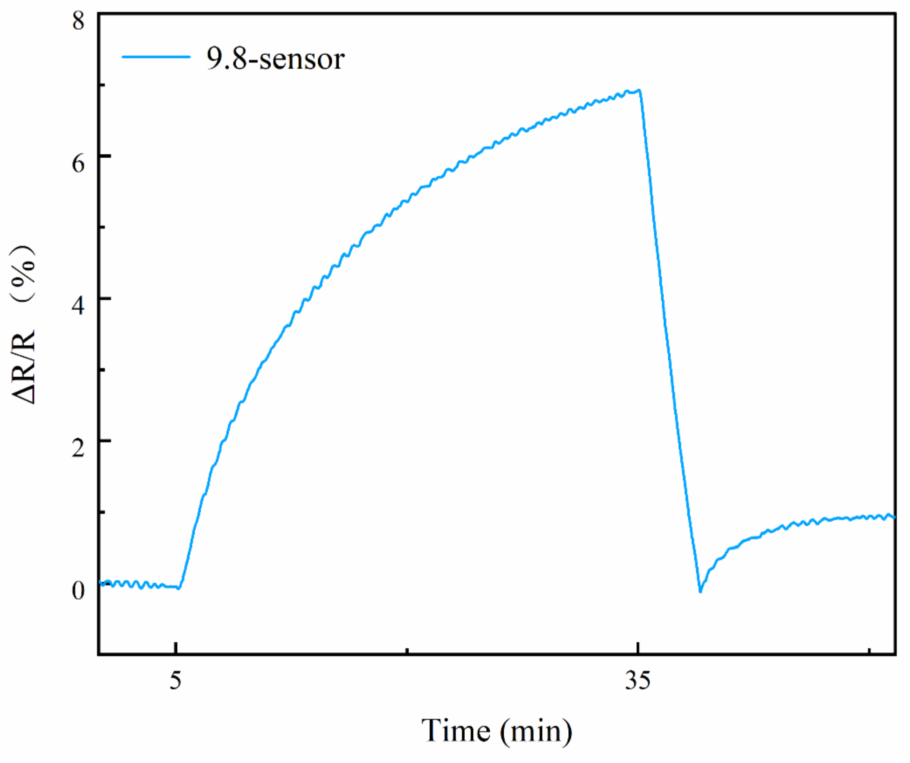

Figure S8. Responsivity of the 9.8-sensor exposed to 0.5 ppm NO_2.

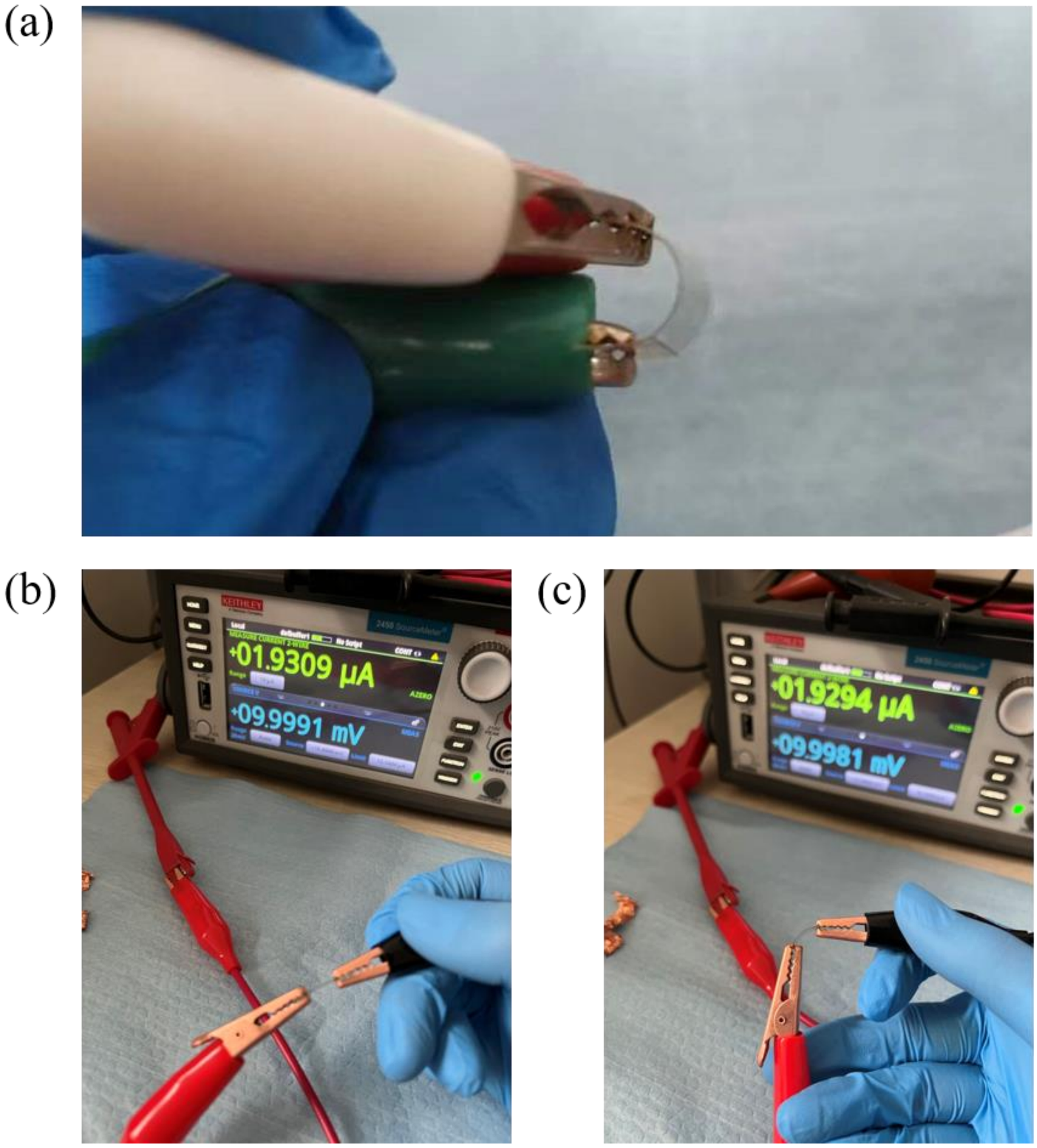

Figure S9. (a) The sensor was bent into a roll with a radius of curvature of 2 mm for 30 times. (b-c) Performance measurement of the sensor after 30 cycles of bending.

Table S1 Parameters of the fluorination process and the F-doping contents.

**Samples**	**XeF_2/CNT**	**Time(h)**	**F(at%)**
1	30	1	0.85
2	30	2	1.19
3	20	4	1.88
4	30	4	2.48
5	20	8	7.4
6	30	8	8.35

(XeF_2/CNT) Mass ratio of pristine XeF_2 to SWCNT. The weight of the CNT film is about 0.1 mg.

Table S2 The contents of pyridinic N (N-6), pyrrolic N (N-5) and graphitic N (N-Q) in N-SWCNT samples calculated from their deconvoluted peak areas.

**Samples**	**N (at%)**	**N-6**	**N-5**	**N-Q**
1	2.9	40.0%	30.7%	29.3%
2	4.3	61.4%	22.6%	16.0%
3	5.6	82.3%	11.0%	6.7%
4	6.4	68.8%	24.2%	7.0%
5	9.8	44.7%	50.3%	5.0%
6	9.9	43.5%	50.3%	6.6%
